# Supplementary material for: Dissemination of pathogenic bacteria is reinforced by a MARTX toxin effector duet
Source: Nat Commun. 2024 Jul 23;15:6218. doi: 10.1038/s41467-024-50650-0 (PMC11266601; doi:10.1038/s41467-024-50650-0)
Supplement: Supplementary file 1 — Supplementary Information [file 41467_2024_50650_MOESM1_ESM.pdf]

Supplementary Information

Dissemination of pathogenic bacteria is reinforced by a MARTX toxin effector duet

Sanghyeon Choi[1,2,11], Youngjin Lee[2,11], Shinhye Park[2,3], Song Yee Jang[2,4], Jongbin Park[2], Do Won Oh[2,5], Su-Man Kim[2,6], Tae-Hwan Kim[2,7], Ga Seul Lee[4,8], Changyi Cho[9], Byoung Sik Kim[9], Donghan Lee[10], Eun-Hee Kim[10], Hae-Kap Cheong[10], Jeong Hee Moon[4], Ji-Joon Song[1], Jungwon Hwang[2,*], and Myung Hee Kim[2,*]

[1]Department of Biological Sciences, Korea Advanced Institute of Science and Technology (KAIST), Daejeon 34141, Korea

[2]Microbiome Convergence Research Center, Korea Research Institute of Bioscience and Biotechnology (KRIBB), Daejeon 34141, Korea

[3]Department of Microbiology and Molecular Biology, Chungnam National University, Daejeon 34134, Korea

[4]Core Research Facility & Analysis Center, KRIBB, Daejeon 34141, Korea

[5]Graduate School of Medical Science and Engineering, KAIST, Daejeon 34141, Korea

[6]Department of Biology Education, Chonnam National University, Gwangju 61186, Korea

[7]College of Veterinary Medicine, Chungnam National University, Daejeon 34134, Korea

[8]College of Pharmacy, Chungbuk National University, Cheongju, Chungbuk 28644, Korea

[9]Department of Food Science and Biotechnology, Ewha Womans University, Seoul 03760, Korea

[10]Korea Basic Science Institute, Cheongju, Chungbuk 28119, Korea

[11]These authors (Sanghyeon Choi and Youngjin Lee) contributed equally to the study.

*Correspondence: jwhwang@kribb.re.kr (J.H.); mhk8n@kribb.re.kr (M.H.K.)

Supplementary Figures 1−14 & Legends

Supplementary Tables 1−2

a

Rank	Protein	Gene	Intensity		Ratio
			Strep	$RID_{C/A}$-Strep	
1	Calmodulin	CALM	5086100	22533000000	4430.3
2	Heat shock 70 kDa protein 1B	HSPA1B	16110000	15266000000	947.6
3	40S ribosomal protein S3	RPS3	ND	13619000000	-
4	Heat shock cognate 71 kDa protein	HSPA8	10026000	11334000000	1130.5
5	Ras-related C3 botulinum toxin substrate 1	RAC1	ND	7001700000	-
6	ADP/ATP translocase 2	SLC25A5	ND	6510100000	-
7	78 kDa glucose-regulated protein	HSPA5	15999000	4795700000	299.7

b

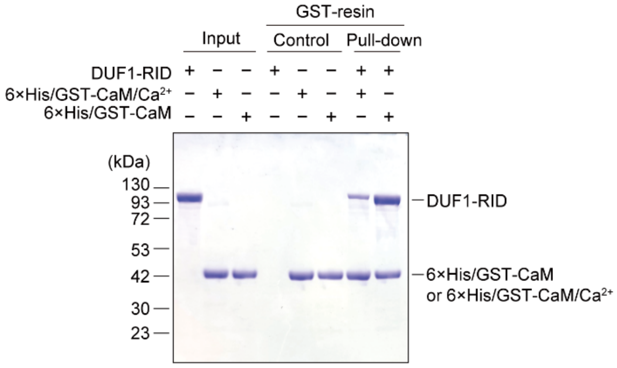

c

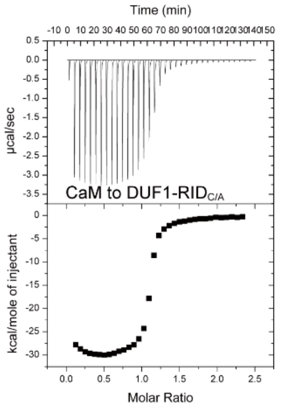

d

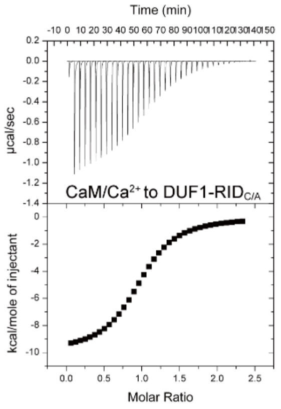

e

Titration	*N*	K_D (μM)
CaM to DUF1-$RID_{C/A}$	1.09 ± 0.00	0.08
CaM/Ca^{2+} to DUF1-$RID_{C/A}$	1.00 ± 0.01	1.93

Supplementary Fig. 1. The DUF1-RID module interacts directly with CaM. a, Potential $RID_{C/A}$-interacting proteins identified by affinity purification mass spectrometry. ND, not detected. **b**, *In vitro* pull-down assay showing direct interaction between the DUF1-RID module and Ca^{2+}-bound or Ca^{2+}-free CaM. **c**, **d**, Raw ITC data showing interactions between DUF1-$RID_{C/A}$ and CaM (**c**), or DUF1-$RID_{C/A}$ and Ca^{2+}-bound CaM (**d**). **e**, ITC results showing binding modes between DUF1-$RID_{C/A}$ and calcium-free CaM or calcium-bound CaM. Data shown in **b**–**e** are representative of three independent experiments, each with similar results. Source data are provided as a Source Data file.

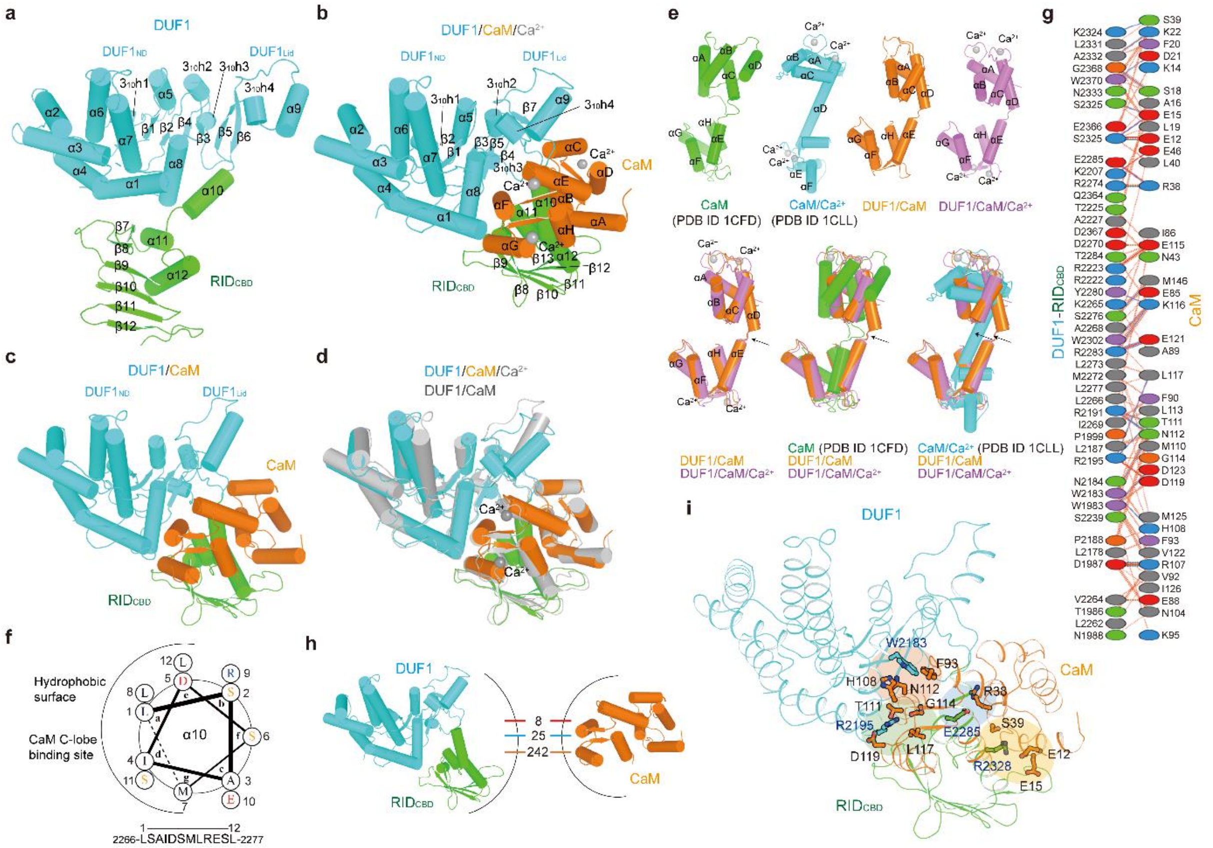

Supplementary Fig. 2. Structural analysis of DUF1-RID$_{CBD}$ and DUF1-RID$_{CBD}$ complexed with CaM. a–c, Overall structures of DUF1-RID$_{CBD}$ (**a**), DUF1-RID$_{CBD}$ complexed with CaM/Ca^{2+} (**b**), and DUF1-RID$_{CBD}$ complexed with CaM (**c**). **d**, Superimposition of DUF1-RID$_{CBD}$ structures complexed with CaM/Ca^{2+} or CaM (gray). **e**, Structural comparison of CaM (PDB ID 1CFD, green) and Ca^{2+}-bound CaM (PDB ID 1CLL, cyan) with that of CaM (orange) and Ca^{2+}-bound CaM (magenta) complexed with DUF1-RID$_{CBD}$. **f**, Helical wheel diagram of the α10 helix in the RID$_{CBD}$. **g**, **h**, Amino acid interaction network between DUF1-RID$_{CBD}$ and CaM. Salt bridges, hydrogen bonds, and hydrophobic contacts are indicated by red, cyan, and orange lines, respectively. **i**, The interaction between DUF1-RID$_{CBD}$ and CaM in detail. DUF1, RID$_{CBD}$, and CaM are shown as cyan, green, and orange cartoon diagrams, respectively. The four residues (W2183, R2195, E2285, and R2328) in DUF1-RID$_{CBD}$ that are critical for the interaction with CaM are indicated.

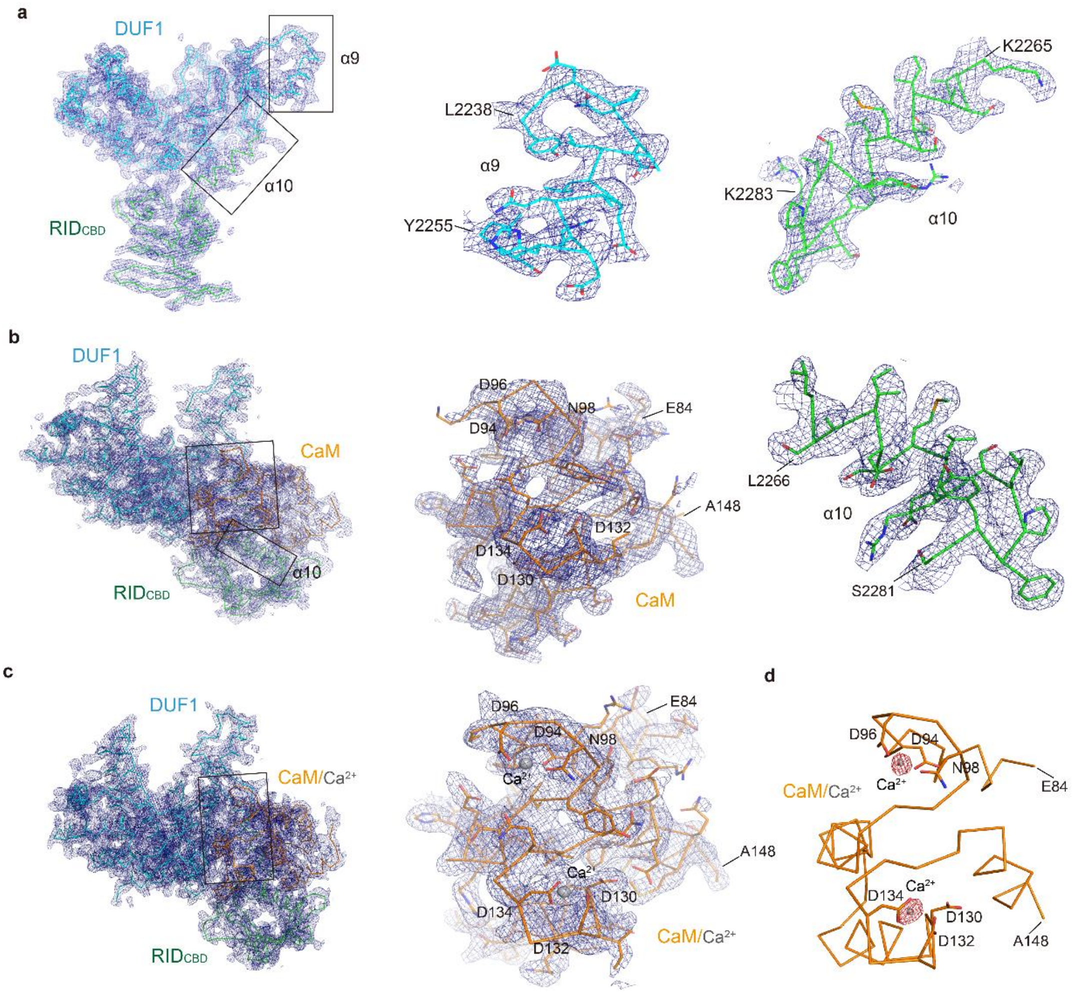

Supplementary Fig. 3. Electron density map of DUF1-RID$_{CBD}$ and DUF1-RID$_{CBD}$ complexed with CaM. **a**, The 2mFo-DFc electron density map of the DUF1-RID$_{CBD}$ is contoured at 1.5 σ level. **b**, **c**, The 2mFo-DFc electron density maps of the DUF1-RID$_{CBD}$ complexed Ca^{2+}-free CaM (**b**) or Ca^{2+}-bound CaM (**c**) are contoured at 1.0 σ level. **d**, The mFo-DFc omit electron density map of Ca^{2+} bound to the CaM complexed with the DUF1-RID$_{CBD}$ is contoured at 4.0 σ level.

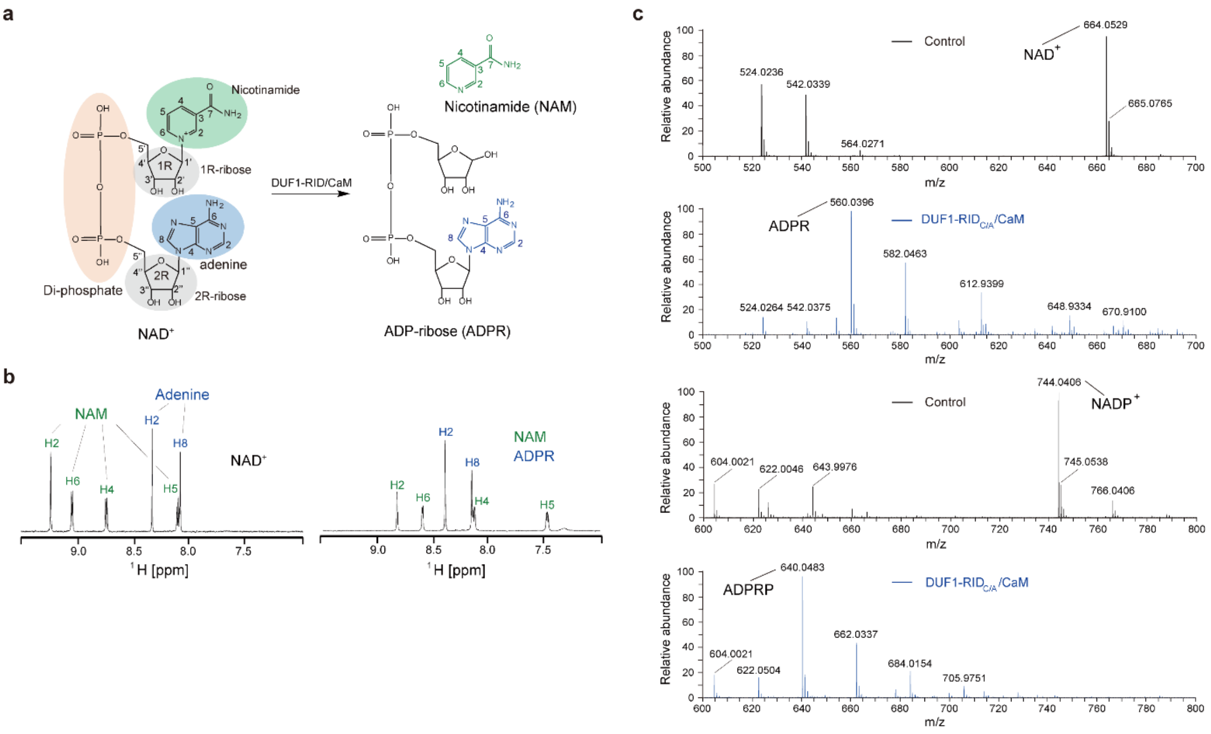

Supplementary Fig. 4. Analysis of by-products resulting from the NAD(P)$^+$-hydrolyzing activity of DUF1-RID. a, Schematics showing hydrolysis of NAD$^+$ to nicotinamide (NAM) and ADP-ribose (ADPR) mediated by DUF1-RID complexed with CaM. The chemical structures of NAD$^+$, NAM, and ADPR are shown, and the hydrogen atoms within NAD$^+$, NAM, and ADPR are numbered. **b**, Assignment of relevant NMR signals to NAD$^+$ and its cleavage products. The protons giving rise to the NMR signals are labeled in green (NAM) and blue (ADPR). 1D proton spectra of NAD$^+$ and its by-products (NAM and ADPR) are shown in the left and right panels, respectively. **c**, MS/MS spectra of NAD(P)$^+$ and its by-products. Fragmentation patterns of NAD$^+$ with m/z = 664.0529 and NADP$^+$ with m/z = 744.0406 are shown in the first and third panels, respectively. The fragmentation patterns of NAD(P)$^+$ by-products (ADPR with m/z = 560.0396 and ADPRP with m/z = 640.0483) following incubation with DUF1-RID$_{C/A}$ complexed with CaM are shown in the second and fourth panels, respectively. Data shown in **b** and **c** are representative of three independent experiments, each with similar results.

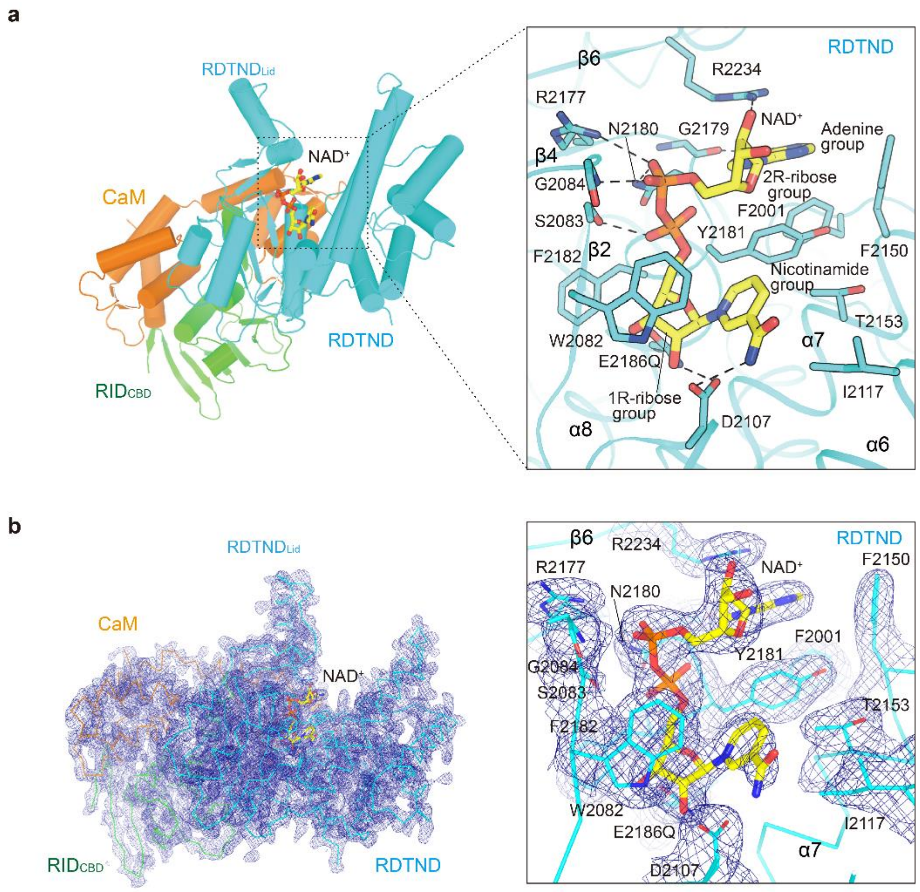

Supplementary Fig. 5. Binding mode of NAD$^+$ at the catalytic site of RDTND-RID$_{CBD}$ complexed with CaM. **a**, Zoom into the catalytic active site of RDTND-RID$_{CBD}$ complexed with CaM. In the magnified panel, NAD$^+$ (yellow) and amino acids (cyan) involved in the interactions are represented by sticks. **b**, The 2mFo-DFc electron density map of the DUF1-RID$_{CBD}$ complexed with CaM and NAD$^+$ is contoured at 1.0 σ level. The mFo-DFc omit electron density map of the NAD$^+$ contoured at 3.0 σ level is shown in Figure 3A.

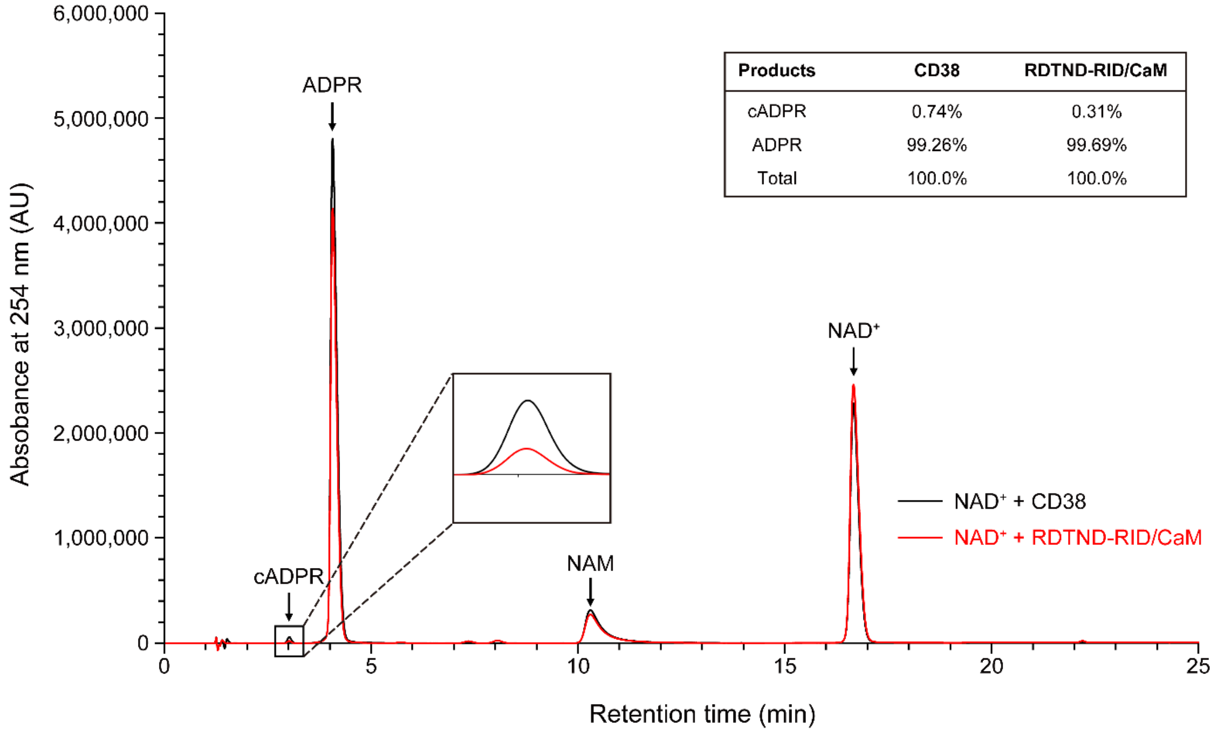

Supplementary Fig. 6. HPLC-UV analysis of NAD^+ byproducts produced by CD38 and RDTND-RID/CaM. The peaks of cADPR production by CD38 (black) and RDTND-RID/CaM (red) are magnified in the box insert. The relative ratios of ADPR and cADPR produced by NAD^+ hydrolysis by CD38 or RDTND-RID/CaM are indicated in the upper right panel. The peaks indicating cADPR, ADPR, NAM, and NAD^+ were analyzed individually by injection of their control compounds (Retention time: cADPR, 3.02 min; ADPR, 4.07 min; NAM, 10.30 min; NAD^+, 16.67 min). Source data are provided as a Source Data file.

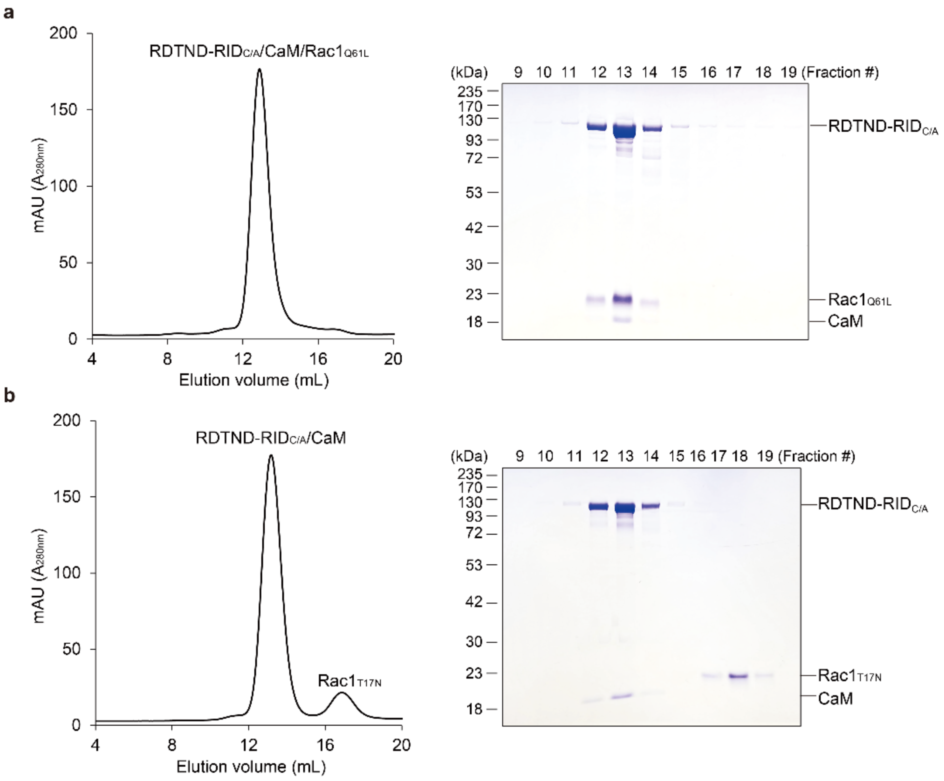

Supplementary Fig. 7. Interaction between the RDTND-RID duet and human target proteins. a, **b**, Size-exclusion chromatography analysis combined with SDS-PAGE demonstrate the interaction between RDTND-$RID_{C/A}$ and human target proteins. The RDTND-$RID_{C/A}$ interaction with CaM and the active mimic Rac1 ($Rac1_{Q61L}$) (**a**), or the inactive mimic Rac1 ($Rac1_{T17N}$) (**b**), was evaluated. Source data are provided as a Source Data file.

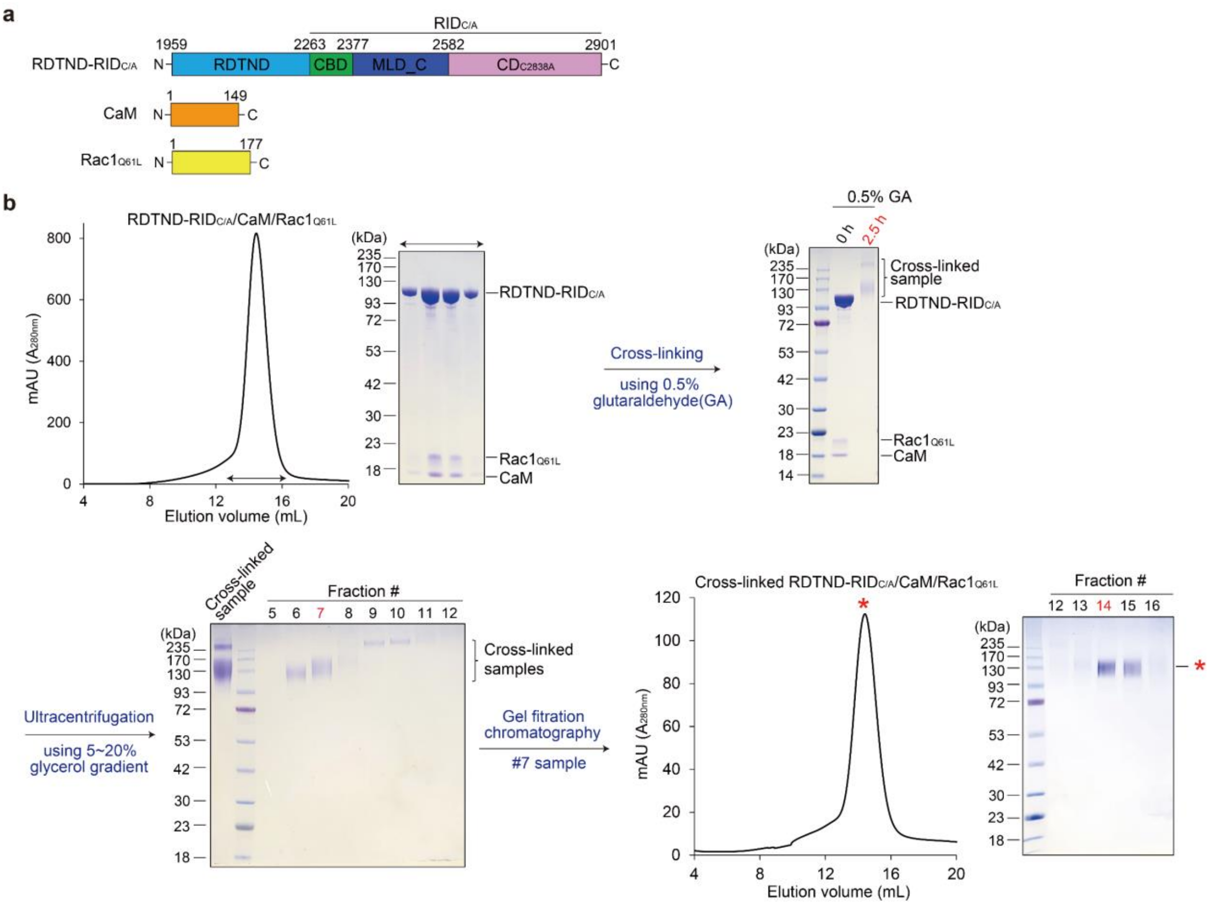

Supplementary Fig. 8. Sample preparation prior to cryo-EM studies of cross-linked RDTND-RID$_{C/A}$ complexed with CaM and Rac1$_{Q61L}$. a, Schematic diagram of the constructs used to purify the recombinant RDTND-RID$_{C/A}$ module complexed with CaM and active mimic Rac1$_{Q61L}$ (RDTND-RID$_{C/A}$/CaM/Rac1$_{Q61L}$). **b,** A flowchart showing purification of the RDTND-RID$_{C/A}$/CaM/Rac1$_{Q61L}$ complex. A detailed description is provided in Methods. Source data are provided as a Source Data file.

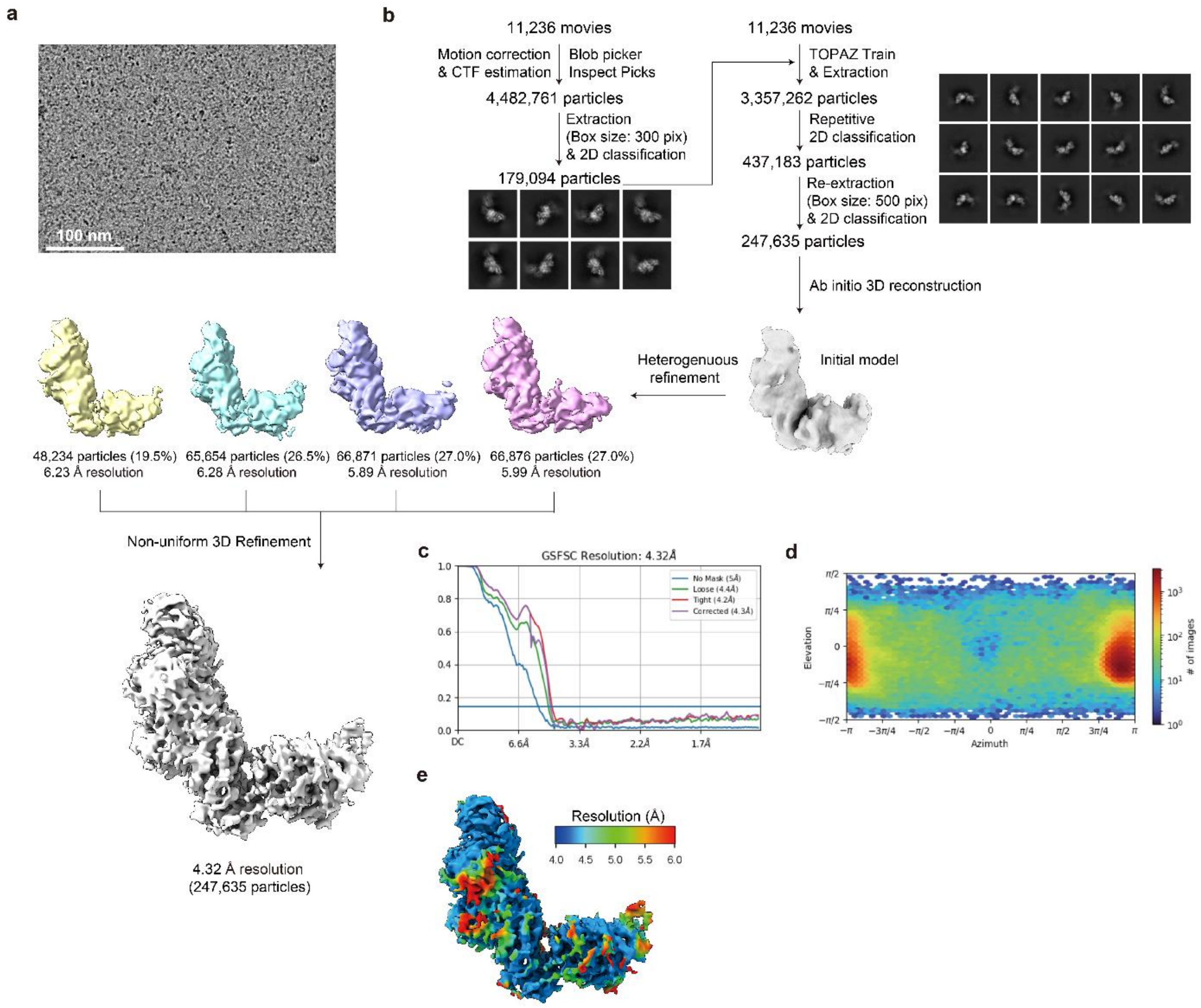

Supplementary Fig. 9. Cryo-EM analysis of cross-linked RDTND-RID$_{C/A}$ complexed with CaM and Rac1$_{Q61L}$. **a**, A representative cryo-EM micrograph showing the complex. Scale bar, 100 nm. **b**, A flowchart showing cryo-EM analysis of the complex. A detailed description is provided in the Methods. **c**, The final reconstruction shows an average resolution of 4.32 Å, as determined by a Fourier shell correlation value of 0.143. **d**, Angular distribution of the complex particles in the final round of non-uniform 3D refinement. **e**, Resolution of the complex, along with color-coded resolution ranges.

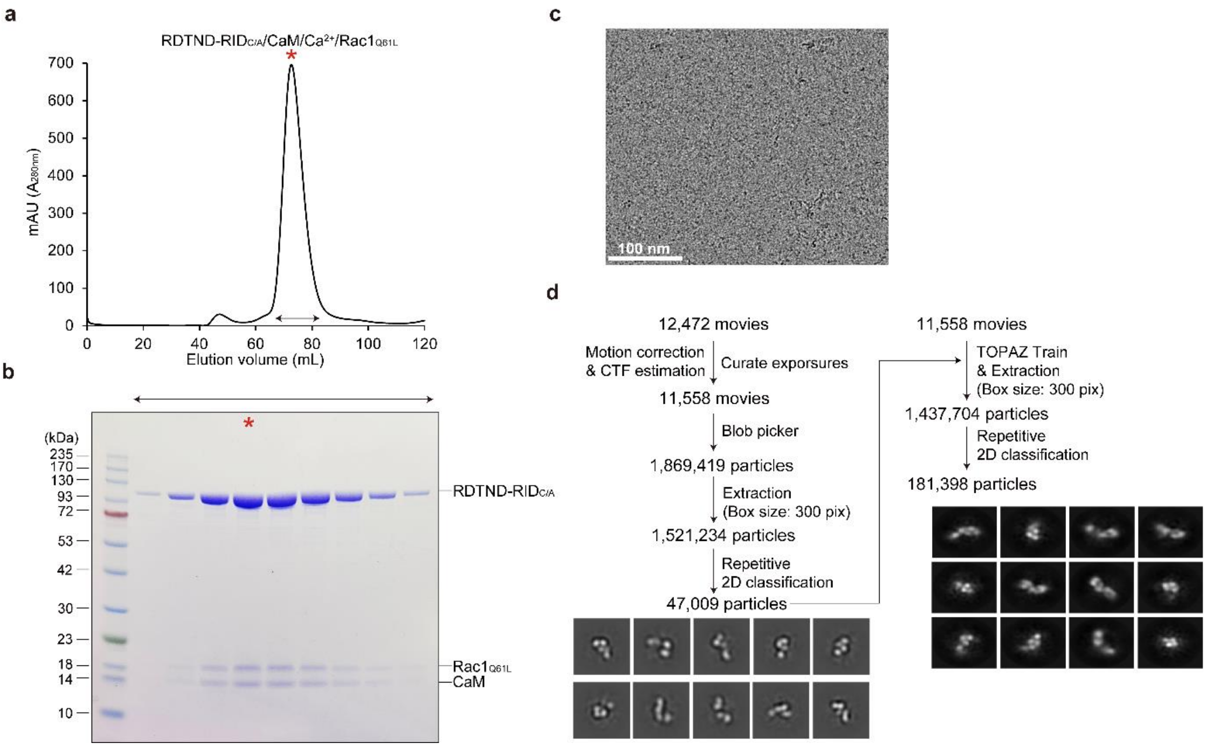

Supplementary Fig. 10. Cryo-EM analysis of RDTND-RID$_{C/A}$ complexed with CaM and Rac1$_{Q61L}$. a, b, Purification of the RDTND-RID$_{C/A}$ complexed with CaM and Rac1$_{Q61L}$. Size-exclusion chromatography analysis (**a**) and SDS-PAGE of the eluted proteins (**b**). **c**, A representative cryo-EM micrograph showing the complex. Scale bar, 100 nm. **d**, A flowchart showing cryo-EM analysis of the complex. Source data are provided as a Source Data file.

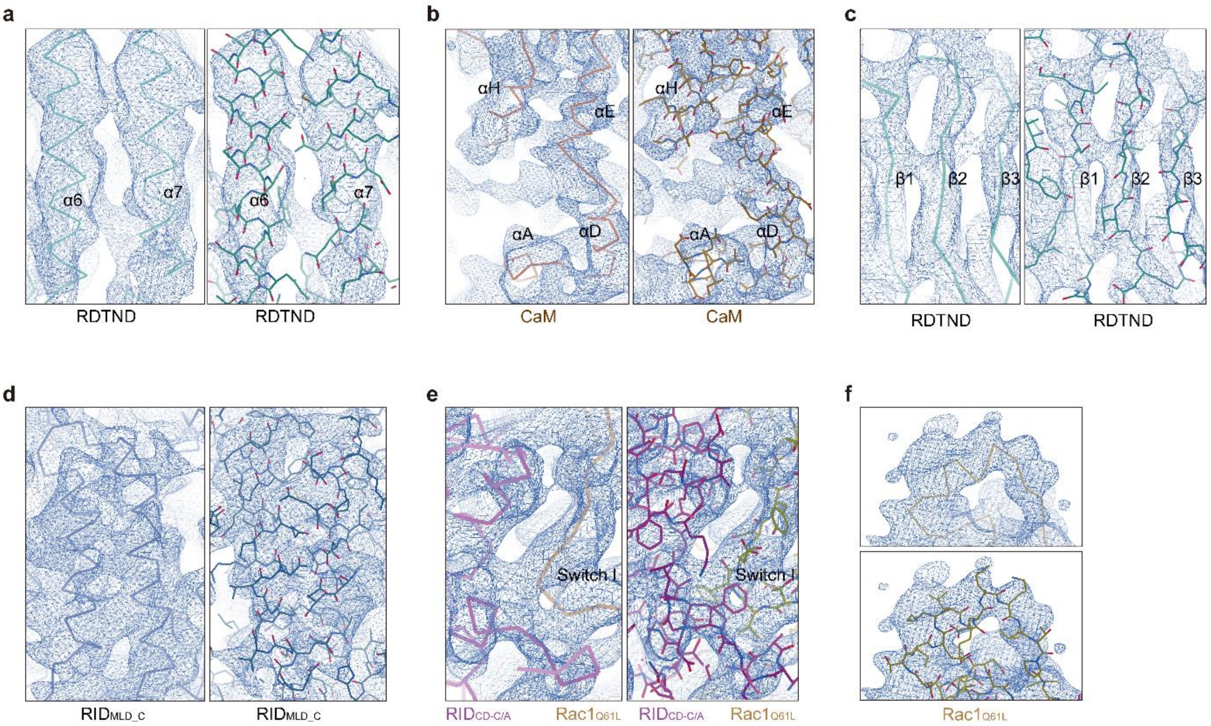

Supplementary Fig. 11. Representative cryo-EM maps of the complex. **a**, Cryo-EM map of RDTND (α6 and α7) contoured at 12 σ. **b**, **c**, Cryo-EM maps of CaM (αA, αD, αE, and αH) (**b**) and RDTND (β1–β3) (**c**) contoured at 8 σ. **d**, Cryo-EM map of the RID_{MLD_C} region (residues 2393–2441) contoured at 6 σ. **e**, Cryo-EM maps of the interface between RID_{CD} (residues 2785–2803) and $Rac1_{Q61L}$ (residues 30–46, Switch I region) contoured at 6 σ. **f**, Cryo-EM map of $Rac1_{Q61L}$ (residues 116–140) contoured at 4.5 σ.

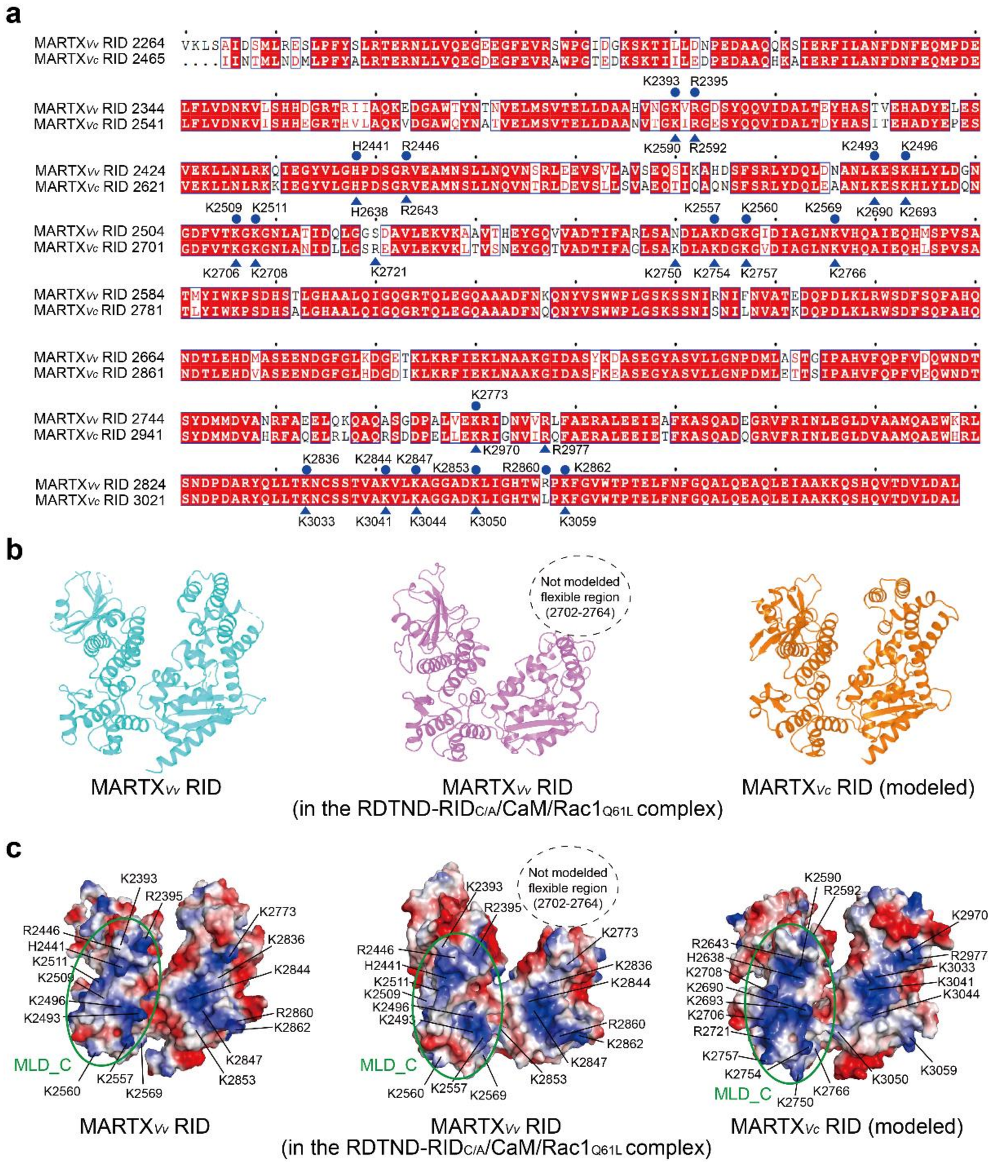

Supplementary Fig. 12. Structural comparison of MARTX*Vv* RID and MARTX*Vc* RID. a, Structure-based sequence alignment of MARTX*Vv* RID (residues 2264–2901, NCBI accession ID: WP_015728045.1) and MARTX*Vc* RID (residues 2465–3098, NCBI accession ID: WP_108347803.1). MARTX*Vv* RID and MARTX*Vc* RID share high amino acid sequence identity (85.6%) and similarity (93.5%). Positive charged residues on surfaces of MARTX*Vv* RID and MARTX*Vc* RID are indicated by blue circles and triangles, respectively. **b**, Overall structures of the MARTX*Vv* RID (PDB ID, 5XN7), MARTX*Vv* RID within the RDTND-RID$_{C/A}$/CaM/Rac1$_{Q61L}$, complex, and MARTX*Vc* RID generated by AlphaFold 3[1]. **c**, Surface charge distribution of the RID proteins. The negative and positive charges on the surface diagrams are shown in red and blue, respectively. MLD_C (i.e., membrane localization domain (MLD)-containing domain) is encircled in green.

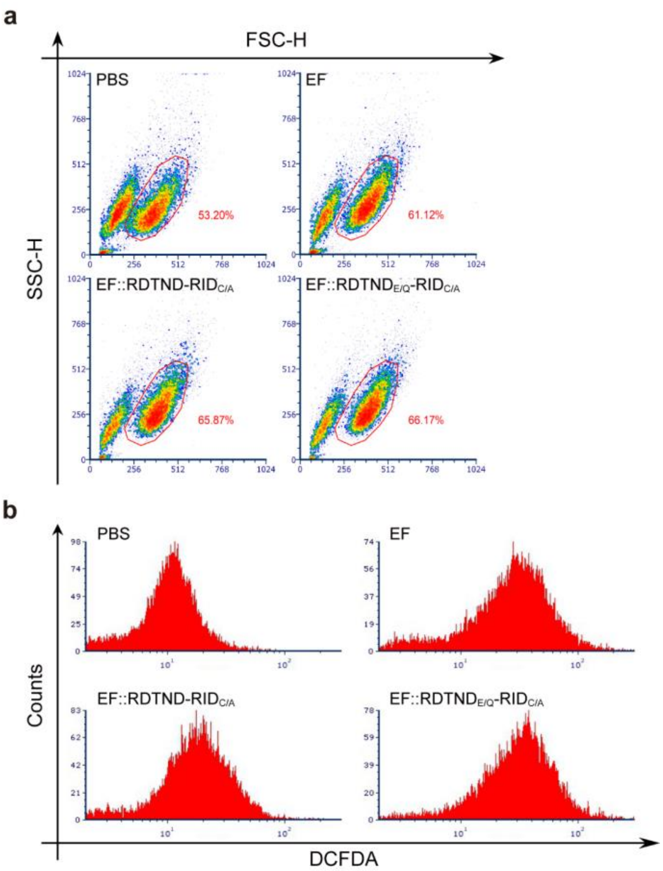

Supplementary Fig. 13. Flow cytometry for ROS detection from RAW 264.7 cells infected with *V. vulnificus* mutant strains. a, RAW 264.7 cells infected with indicted *V. vulnificus* strains were analyzed via flow cytometry using forward-scatter height (FSC-H) and side-scatter height (SSC-H). Proportions of cell counts within the defined polygon gate are indicated. **b**, ROS levels in RAW 264.7 cells infected with indicated *V. vulnificus* strains were detected using DCFDA and presented as histograms.

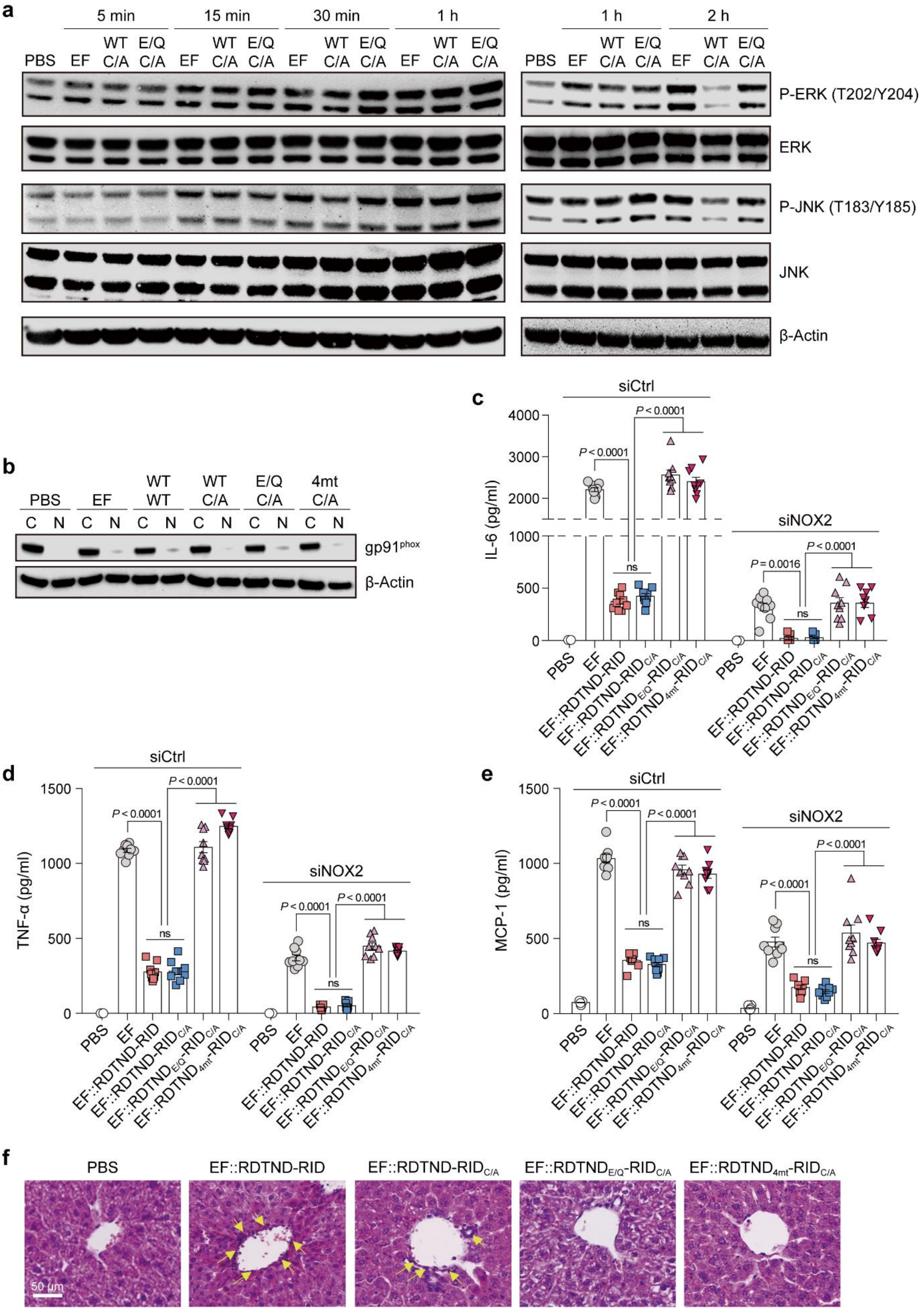
a
5 min
15 min
30 min
1 h
2 h
PBS
EF
WT C/A
E/Q C/A
P-ERK (T202/Y204)
ERK
P-JNK (T183/Y185)
JNK
β-Actin
b
PBS
EF
WT WT
WT C/A
E/Q C/A
4mt C/A
C
N
gp91phox
β-Actin
c
siCtrl
siNOX2
IL-6 (pg/ml)
P < 0.0001
P = 0.0016
ns
PBS
EF
EF::RDTND-RID
EF::RDTND-RIDC/A
EF::RDTNDE/Q-RIDC/A
EF::RDTND4mt-RIDC/A
d
TNF-α (pg/ml)
e
MCP-1 (pg/ml)
f
50 μm

Supplementary Fig. 14. Immunological analyses of immune cells and mice infected with *V. vulnificus* mutant strains. **a**, Immunoblot assay of MAPK signaling in infected BMDMs. BMDMs were treated with PBS for 2 h, or infected with *V. vulnificus* strains (MOI = 5, 5 × 10^6 CFU) for the indicated times. **b**, Immunoblot analysis of cell lysates from RAW 264.7 cells treated with PBS or infected with indicated *V. vulnificus* strains for 2 h. EF, effector-free; WT-C/A, EF::RDTND-$RID_{C/A}$; E/Q-C/A, EF::$RDTND_{E/Q}$-$RID_{C/A}$ strain; C, non-targeting siRNA treated; N, $gp91^{phox}$-targeting siRNA treated. **c**–**e**, ELISA analysis of pro-inflammatory cytokines IL-6 (**c**), TNF-α (**d**), and MCP-1 (**e**) in supernatants from *V. vulnificus*-infected RAW 264.7 cells transfected with $gp91^{phox}$-specific siRNA (siNOX2) or non-targeting siRNA (siCtrl). Data are presented as the mean ± SEM (n = 9 per group). P-values were calculated using two-way ANOVA with multiple comparisons. **f**, Histological analysis of H&E-stained tissue sections of liver collected from mice infected with the indicated *V. vulnificus* strains (n = 3 per group). Yellow arrows indicate infiltrated immune cells around blood vessels in the liver. Data shown in **a**, **b**, and **f** are representative of three independent experiments, each with similar results. Source data are provided as a Source Data file.

Supplementary Table 1. Data collection and refinement statistics

	$RDTND\text{-}RID_{CBD}$	$RDTND\text{-}RID_{CBD}/CaM/Ca^{2+}$		$RDTND_{E/Q}\text{-}RID_{CBD}/CaM/Ca^{2+}/NAD^{+}$	$RDTND\text{-}RID_{CBD}/CaM$
**Crystal data**	Se-Met	Native	Se-Met	Native	Se-Met
**Data collection**					
Space group	*P*6	*C*2	*C*2	$P2_1$	$P2_1$
X-ray source[a]	PAL-5C	PAL-5C	PAL-5C	PAL-5C	PAL-11C
Detector	Dectris Eiger X 9M	Dectris Eiger X 9M	Dectris Eiger X 9M	Dectris Eiger X 9M	Dectris Pilatus3 6M
	Peak	*Native*	*Peak*	*Native*	*Peak*
Wavelength (Å)	0.9795	0.9797	0.9795	1.0000	0.9794
Unit cell					
a (Å)	207.73,	243.37	243.80	136.22	106.03,
b (Å)	207.73,	49.78	49.37	48.93	88.08,
c (Å)	54.65	135.75	136.09	178.60	136.95
α (°)	90.00,	90.00	90.00	90.00	90.00,
β (°)	90.00,	119.35	119.48	95.70	90.14,
γ (°)	120.00	90.00	90.00	90.00	90.00
Resolution range (Å)[b]	50.00–3.38 (3.44–3.38)	50.00–2.90 (2.95–2.90)	50.00–3.05 (3.10–3.05)	177.72–2.35 (2.39–2.35)	50.00–2.82 (2.87–2.82)
R_{merge}[c]	5.7 (154.1)	6.3 (127.6)	6.0 (123.1)	10.8 (127.1)	13.2 (110.5)
$C/C^{1/2}$	99.6 (56.8)	99.5 (58.1)	99.7 (54.4)	99.7 (67.5)	98.8 (56.1)
$I/\sigma I$	11.1 (1.2)	9.3 (1.2)	9.7 (1.3)	11.6 (1.6)	5.3 (1.2)
Completeness (%)	99.6 (99.6)	99.3 (97.8)	98.4 (94.2)	99.8 (99.9)	98.1 (98.0)
Redundancy	6.2 (5.6)	6.3 (5.9)	6.4 (5.7)	6.9 (7.2)	4.7 (4.6)
**Refinement**					
Resolution range (Å)	40.42–3.38	48.51–2.90	–	29.62–2.35	49.53–2.82
No. reflections	15125	26461	–	93729	51961
R_{work}[d] (%) / R_{free} (%)	28.60 / 32.21	26.50 / 30.40	–	21.74 / 26.35	27.14 / 34.15
No. atoms / residues	5848 / 729	8608 / 1099	–	18350 / 2830	16066 / 2157
Protein	5848 / 729	8586 / 1077	–	17526 /2198	16045 / 2136
Ion (Ca^{2+})	–	8 / 8	–	8 / 8	3 / 3
NAD	–	–	–	176 / 4	–
Glycerol	–	–	–	24 / 4	–
Water	–	14 / 14	–	616 / 616	18 / 18
B-factors (Å^2)					
Protein	59.3	49.4	–	58.9	55.5
Ca^{2+}	–	67.8	–	63.0	–
Mg^{2+}					41.1
NAD	–	–	–	55.3	–
Glycerol	–	–	–	65.1	–
Water	–	20.7	–	47.0	29.4
R.m.s. deviations					
bond length (Å)	0.009	0.016	–	0.010	0.013
bond angles (°)	1.312	1.102	–	1.033	1.044
Ramachandran plot					
Favored (%)	93.0	94.5	–	94.8	93.8
Allowed (%)	7.0	5.5		5.2	6.2
Disallowed (%)	0	0		0	0

[a]Beamline 5C and 11C at Pohang Acceleratory Laboratory (PAL) in South Korea

[b]Values in parentheses are for the highest-resolution shell.

[c]$R_{merge} = \sum_h \sum_i |I(h)_i - \langle I(h)\rangle| / \sum_h \sum_i I(h)_i$, where I(*h*) is the intensity of reflection of *h*, $\sum_h$ is the sum over all reflections and $\sum_i$ is the sum over *i* measurements of reflection *h*.

[d]$R_{work} = \Sigma_{hkl} ||F_o|-|F_c|| / \Sigma_{hkl}|F_o|$; 5% of the reflections were excluded for the R_{free} calculation.

r.m.s deviations, root mean square deviations

Supplementary Table 2. Cryo-EM data collection and processing statistics

	RDTND-RID$_{C/A}$/ CaM/Rac1$_{Q61L}$ (EMD-37593)
**Data collection and processing**	
Magnification	130,000
Voltage (kV)	300
Electron exposure (e–/Å^2)	50
Defocus range (μm)	-1.4 ~ -2.2
Pixel size (Å)	0.66
Symmetry imposed	C1
Initial particle images (no.)	3,357,262
Final particle images (no.)	247,635
Map resolution (Å)	4.32
FSC threshold	0.143
Map resolution range (Å)	4.32−4.92
**Refinement**	-
Initial model used (PDB code)	-
Model resolution (Å)	-
FSC threshold	
Model resolution range (Å)	-
Map sharpening *B* factor (Å^2)	-
Model composition	-
Non-hydrogen atoms	
Protein residues	
Ligands	
B factors (Å^2)	-
Protein	
Ligand	
R.m.s. deviations	-
Bond lengths (Å)	
Bond angles (°)	
Validation	-
MolProbity score	
Clashscore	
Poor rotamers (%)	
Ramachandran plot	-
Favored (%)	
Allowed (%)	
Disallowed (%)	

Supplementary References

1 Abramson, J. *et al.* Accurate structure prediction of biomolecular interactions with AlphaFold 3. *Nature* **630**, 493-500, doi:10.1038/s41586-024-07487-w (2024).
