# Supplementary material for: Dissemination of pathogenic bacteria is reinforced by a MARTX toxin effector duet
Source: Nat Commun. 2024 Jul 23;15:6218. doi: 10.1038/s41467-024-50650-0 (PMC11266601; doi:10.1038/s41467-024-50650-0)
Supplement: Supplementary file 3 — Description of Additional Supplementary Files [file 41467_2024_50650_MOESM3_ESM.pdf]

Description of Additional Supplementary Files

File Name: Supplementary Data 1
Description: Experimental materials used in this study

File Name: Supplementary Data 2
Description: Primers and plasmids used in this study

File Name: Supplementary Data 3
Description: Structural model of the RDTND-RID$_{C/A}$/CaM/Rac1$_{Q/L}$ complex fitted into cryo-EM density map

File Name: Supplementary Data 4
Description: Interactome analysis for identification of RID-interacting candidates
